# The Antioxidant Properties and Protective Capacity of *Prangos trifida* and *Cachrys cristata* Essential Oils against Cd Stress in *Lunularia cruciata* and *Brassica napus*

**DOI:** 10.3390/antiox12040793

**Published:** 2023-03-24

**Authors:** Viviana Maresca, Natale Badalamenti, Vincenzo Ilardi, Maurizio Bruno, Adriana Basile

**Affiliations:** 1 Dipartimento di Biologia, Università Degli Studi di Napoli Federico II, Complesso Universitario Monte Sant’Angelo, Via Cinthia 4, 80126 Napoli, Italy; 2 Dipartimento di Scienze e Tecnologie Biologiche, Chimiche e Farmaceutiche (STEBICEF), Università degli Studi di Palermo, Viale delle Scienze Ed. 17, 90128 Palermo, Italy; 3 NBFC, National Biodiversity Future Center, 90133 Palermo, Italy

**Keywords:** *Cachrys* L., *Prangos* Lindl., essential oils, *Lunularia cruciata*, *Brassica napus*, antioxidant enzymes, DNA damage

## Abstract

The genera *Prangos* Lindl. and *Cachrys* L., for a long time interpreted as a single genus but today distinct and separate, and both belonging to the majestic Apiaceae family, are species with a large distribution and are used in ethnomedicine in various countries, especially in Asian countries. In this context, we investigated the chemical characteristics and biological properties of two essential oils (EOs) obtained from different specimens, namely *Cachrys cristata* (Cc) and *Prangos trifida* (Pt). The chemical composition of the two EOs was investigated by GC-MS analysis. From gas-chromatography analyses, while the (Cc) EO was rich in *β*-myrcene (45.34%), *allo*-ocimene (10.90%), and 2,4,6-trimethylbenzaldehyde (23.47%), the (Pt) EO was characterized by moderate amounts of *α*-pinene (8.85%), sylvestrene (11.32%), *α*-phellandrene (12.14%), (*Z*)-*β*-ocimene (18.12%), and finally, *p*-mentha-1,3,8-triene (9.56%). Furthermore, the protective and antioxidant capacity of (Pt) and (Cc) EOs on *Lunularia cruciata* and *Brassica napus* exposed to cadmium (Cd) stress was studied. To study these possible effects, the liverwort and oilseed rape, previously pretreated with both EOs, were subsequently subjected to oxidative stress by treatment with Cd. Then, DNA damage and antioxidant enzyme activity were measured in both EOs-pretreated and untreated samples to examine EOs-induced tolerance to Cd toxicity. The results indicate that (Pt) and (Cc) EOs have antioxidant and protective capacity in modulating the redox state through the antioxidant pathway by reducing oxidative stress induced by Cd. Furthermore, *B. napus* was found to be a more resistant and tolerant species than *L. cruciata.*

## 1. Introduction

Cadmium is an ecologically dangerous toxic metal that can cause harm to the health of living organisms. Cadmium (Cd), together with lead (Pb), arsenic (As), and mercury (Hg), is considered an element with a high degree of toxicity, responsible for many types of damage to human health [1,2]. Despite the extreme toxicity of Cd, exposure to it is constantly increasing [3]. Generally, the route through which Cd is taken up from the environment is through the ingestion of water or food; it then reaches the bloodstream, and through the absorption process it causes damage to various tissues and organs such as the pancreas, liver, etc. Cd’s harmful action derives from stimulating the formation of free radicals and reactive oxygen derivatives, which cause lipid peroxidation and oxidative stress [4,5]. The chronic oral minimum risk value regulated by the EPA (Environmental Protection Agency), United States, Washington, for adults is 1 µg/kg/day for Cd. Due to the difficulty in quantifying the effects of heavy metals, and due to the low (trace) concentrations in different human biological matrices, biological monitoring of human exposure to heavy metals has become challenging. For this reason, it has become increasingly widespread and indispensable to carry out environmental monitoring that uses model organisms, which are easier to find as bioindicators of environmental pollution than plants. Plants growing in the presence of Cd show biochemical and physiological disorders such as the impaired activity of different biological systems, also implicating cell death [6]. This leads to an increase in reactive oxygen species (ROS)—the main cause of lipid and protein oxidation—as well as damage to DNA and mRNA and harmful interactions with different plant cellular structures [7].

However, phytotoxic concentrations of Cd are very different between plants and depend on species, landraces, and cultivars [8]. Some plants can tolerate heavy metal pollution better than others. A plant equipped with a good antioxidant system that has different antioxidant components can eliminate excess ROS and thus protect cells from oxidative damage [9].

Recently, attention is being paid to the protective capacity of essential oils (EOs) against heavy metal stress [10]. In nature, EOs play an important role in plant protection by virtue of their antibacterial, antiviral, antifungal, insecticidal, and antioxidant properties. EOs are volatile natural compounds of complex composition, fat-soluble and soluble in organic solvents, have a density generally lower than that of water, are characterized by a strong odor, are obtained from aromatics as secondary metabolites, and are mainly obtained from aromatic plants.

After its establishment, the genus *Cachrys* L. underwent numerous—often contradictory—nomenclatural and taxonomic rearrangements that were the cause of considerable interpretative confusion that is still very topical today. The genus is made up of perennial herbaceous plants up to 150 cm tall; the leaves (2–6) are pinnate, with rhombic or triangular outlines. It has composite umbels with bracts and bracteoles, simple or pinnate. Seeds have concave endosperm and are involute on the commissural face [11]. In accordance with Gruenberg-Fertig et al. [12] and Pimenov & Tikhomirov [13], the peculiar anatomical structures possessed by the fruits, observable and characterizable with the use of microscopy, are considered fundamental for the characterization and distinction of the genera *Cachrys* L. and *Prangos* Lindl.

The classification of the *Cachrys* and *Prangos* genera has been somewhat confused. In fact, according to some authors, *Cachrys* genus also includes *Prangos* Lindley and *Hippomarathrum* Link, whereas other authors have classified them into three different genera. Currently, this classification is the accepted one [14]. Two recent reviews have been published on genus *Cachrys* [14] and genus *Prangos* [15], both summarizing the phytochemical compositions of non-volatile and volatile metabolites, their uses in traditional medicine in different countries, and their biological properties.

*Cachrys cristata* DC. (**Cc**), found and collected in Crete, is the only representative of this genus on this island [16]. The species seems to have a distribution limited to the central and eastern portions of the Balkan Peninsula, including Greece, the eastern Aegean Islands, Crete Island, and Asiatic Turkey. The western limit of the range seems to be made up of southern Italy (Puglia and Calabria) and the islands, limited to Sicily [17]. It is a glabrous perennial herbaceous species, with leaves arranged alternately at the base, then progressively opposite and coiled in the upper part of the stem. Flowers, radially symmetrical, are yellow, from 3 to 3.5 mm in diameter, united in a composite umbel. The 7–10 mm fruits have dentate crested, almost winged ribs [18,19].

The perennial plant *Prangos trifida* (Mill.) (Pt) Herrnst. & Heyn, widespread in Spain, France, northern Italy, and Bulgaria, has stems of 50–120 cm, glabrous and solid, with large basal leaves (40 × 40 cm, 4–7 pinnatisect) [20]. Only two publications have dealt with the phytochemical aspects of (Pt). The first one highlighted the presence of furanocoumarins such as imperatorin, isoimperatorin, and prantschimgin; while the second dealt with the chemical composition of the EOs isolated from four different stations collected in Spain, and the results are discussed later [21].

On the other hand, investigation of the non-volatile metabolites of a Turkish accession of *C. cristata* showed the occurrence of (+)-peucadanol methyl ether (ulopterol) [22], whereas several papers have been published on the EO of this species [23,24,25,26], and their results will be commented on later.

In the framework of our recent research on antioxidant [27,28,29], antibacterial [28,29,30], and biocidal [31] properties of EOs from the Apiaceae family, the protective and antioxidant capacity of (Pt) and (Cc) EOs has been tested. To study these possible effects, two “model” plants, *Lunularia cruciata* and *Brassica napus*, pretreated with both EOs, were subsequently subjected to oxidative stress by treatment with Cd. Then, DNA damage and antioxidant enzyme activity were measured in both EOs-pretreated and EOs-untreated samples to examine EOs-induced tolerance to Cd toxicity.

## 2. Materials and Methods

### 2.1. Essential Oil Isolation

The aerial parts of (**Cc**) were collected along the road from Omalos to Pelekanos Paleochora, Crete, Greece (35°14′54″ N, 23°48′34″ E, 500 m m.s.l.), in June 2022 and a voucher specimen was deposited in STEBICEF Department, University of Palermo, Palermo, Italy (Voucher No. PAL 113455). Aerial parts from twenty individuals, covering about 200 m^2^ of (Pt), were harvested at Rocchetta Nervina, Liguria, Italy, 300 m m.s.l. (43°53′21.45″ N; 7°36′02.29″ E), in June 2022. A part of the collected samples, identified by Vincenzo Ilardi, were deposited in Department, University of Palermo, Palermo, Italy (Voucher No. PAL 113456).

Both plant materials (≈200 g) of (Cc) and (Pt) were frozen immediately after harvesting, and once defrosted were mixed with about 500 mL of water and subjected to hydrodistillation for 3 h using Clevenger’s apparatus [32]. The EOs, with yields of 0.65% and 0.30% (*w*/*w*) respectively, were dried with anhydrous sodium sulphate, filtered, and stored in the freezer at −20 °C until the time of analysis.

### 2.2. GC-MS Analysis of Essential Oils

Analyses of both EOs were performed according to the procedure reported by Rigano et al. [31]. One μL of diluted EOs [2/100 *v*/*v*, in *n*-hexane (Sigma-Aldrich St Louis, MO, USA)] was injected into an Agilent 7000 C GC (Agilent, Santa Clara, CA, USA) system equipped with a split/splitless injector and a GERSTEL automatic sampler (GERSTEL, Linthicum, MD, USA). The column set was composed of a fused silica Agilent DB-Wax capillary column (30 m × 0.25 mm I.D.; 0.25 μm film thickness, Agilent, Santa Clara, USA). All details of the method and the identification of the compounds are reported in Rigano et al. 2020 [33].

### 2.3. Plant and Heavy Metal Treatment

#### 2.3.1. *Brassica napus*

Rape seeds (*Brassica napus* L.) were sterilized with a NaClO solution (0.1%) and planted in pots filled with perlite in a growth chamber with a day/night temperature of 25/20 °C and 16 h of light/dark photoperiod of 8 h, with 70% relative humidity. Plants irrigated with distilled water and Hoagland’s nutrient solution (pH 5.7 ± 0.1) were treated with 0.25% (*v/v*) EO of (Pt) and (Cc) as a foliar spray for 7 days. EOs were dissolved in 5% dimethyl sulfoxide (DMSO) followed by dilution with water containing the surfactant Tween 20 (0.1%, *v*/*v*).

For applying the EO test samples, 4 mL of each test sample solution was sprayed onto each pot at the same time. After pretreatment, the plants were irrigated with Hoagland’s solution containing 1.5 mM CdCl_2_ for 7 days.

Six plants were used for controls [without pretreatment with EOs of (Pt) and (Cc), and without treatment with CdCl_2_], six plants were exposed to treatment with CdCl_2_ but without pretreatment with EOs, and six plants were pretreated with EOs and exposed to CdCl_2_ treatment. At the end of experiments, leaves from treated and untreated (control) samples were collected for analysis.

#### 2.3.2. *Lunularia cruciata*

The liverwort *L. cruciata* L. (Dum.) was collected from the Riccia countryside (Campobasso, Molise, Italy 41.493698° N, 14.833967° E) in a hilly area far from known sources of pollution, and it was verified through different methods of analysis that there was an absence of heavy metal pollution. Three grams of the samples, cleaned with distilled water, were inoculated into flasks containing sterile modified Mohr’s medium [34] and cultured for 21 days (acclimatization). Liverwort was placed in Petri dishes so that Mohr’s solution wetted only the lower portion of the thallus. It was important not to “submerge” the samples to ensure that the plant was able to carry out gas exchange through the pores correctly. Then, EOs of (Pt) and (Cc) were applied as foliar spray (see Section 2.3.1) on the gametophytes for 7 days. Then, the plants pretreated with and without EOs were irrigated with Mohr solution containing 1.5 mM CdCl_2_ for 7 days in a climatic room with a temperature ranging from 13 to 20 °C (night/day), 70% relative humidity, and a photoperiod of 16 h light (40 *μ*Em^−2^s^−1^ intensity) and 8 h dark. Six samples were used for each treatment (see Section 2.3.1).

### 2.4. Detection of ROS and Antioxidant Activity Enzyme

The samples of *B. napus* and *L. cruciata* were homogenized with Phosphate Buffered Saline (PBS) solution (Sigma-Aldrich) (0.1 mL of 50 mM, pH 7.4) using a sterile pestle. The protein extract was used to evaluate the levels of ROS and the activity of the antioxidant enzymes.

ROS levels were assessed using 2′,7′-dichlorofluorescin diacetate (H2DCFDA). The extract was incubated with 5 μM H2DCFDA for 30 min at 37 ± 1 °C. ROS quantity was monitored by fluorescence (excitation wavelength of 350 nm and an emission wavelength of 600 nm).

The activity of the antioxidant enzymes catalase (CAT) (Sigma-Aldrich Co., St Louis, MO, USA), Superoxide dismutases (SOD) (19160, Sigma, St Louis, MO, USA), and glutathione S-transferases (GST) (CS0410, Sigma St Louis, MO, USA) were measured following the kit instructions. The level of ROS and the antioxidant activity enzyme was detected using a microplate reader (Bio-Rad Laboratories Inc., Hercules, CA, USA) [35]. For each sample, 3 replicates were performed.

### 2.5. Comet Assay

Six samples for each treatment (see Section 2.3.1) were sliced using a fresh razor blade. The plate was kept tilted on ice so that the isolated nuclei would collect in cold Tris buffer. The protocol was performed as reported by Maresca et al. [35].

### 2.6. Statistical Analysis

ROS production and SOD, CAT, and GST enzyme activities were examined by one-way analysis of variance (ANOVA) and Tukey’s test. In all Figures, values are presented as mean ± st. err; numbers not accompanied by the same letter are significantly different at *p* < 0.05. Statistica software was used to analyze all data (Statistica ® 7.0, StatSoft 7.0, Tulsa, OK, USA) [10].

## 3. Results and Discussion

### 3.1. Gas Chromatography and Mass Spectrometry (GC-MS) Analysis of the Essential Oils

Hydro-distillation of the aerial parts of (Pt) gave a pale-yellow oil with a yield of 0.30% (*w/w*), and its composition has been recently reported [36]. The oil was particularly rich in monoterpene hydrocarbons (71.26%), with *cis*-*β*-ocimene (18.12%), *α*-phellandrene (12.14%), sylvestrene (11.32%), *p*-mentha-1,3,8-triene (9.56%), and α-pinene (8.85%) as main constituents. The second most abundant class was represented by sesquiterpene hydrocarbons (16.74%), with germacrene D (5.53%) and zingiberene (5.32%) as principal metabolites. On the other hand, oxygenated monoterpenes (1.68%), oxygenated sesquiterpenes (1.31%), and oxygenated diterpenes (0.31%) were present in almost negligible amounts (Table 1).

Comparing these results with those obtained by chemical investigations of EOs obtained from different parts of (Pt) collected in Spain (Rivas-Vaciamadrid and Los Santos de Humosa) [21], it is possible to highlight how they contained consistent quantities of *cis-β*-ocimene (20.50–51.50%), a chemical compound totally absent in the plants collected in the other localities of Chinchón and Colmenar de Oreja (Spain). Furthermore, the other two metabolites present, namely α-phellandrene and sylvestrene, were present only in small amounts in all four Spanish accessions [21]. It should also be noted that limonene and *γ*-terpinene, among the main constituents of Spanish plants, were not present in our EO. On the other hand, the composition of the (Pt) EO collected in Serbia was totally different. The EOs of its areas, analyzed separately (leaves, stems, and fruits), were found to be rich in terpinolene (18.1%), *p*-cymen-8-ol (21.8%), *p*-cymene (14.1–25.4%), limonene (14.4%), and (*E*)-*β*-ocimene (23.2%), while the EO analyzed in this work had higher quantities of the isomer (*Z*)-*β*-ocimene (18.12%) [37].

The GC-MS analysis on a DB-Wax polar column of the EO obtained from the aerial parts of (Cc) (Table 1) allowed the identification of fourteen compounds, accounting for 97.62% of the total composition. Also, in this case, the main class was represented by monoterpene hydrocarbons (67.63%), with the most abundant components being *β*-myrcene (45.34%), *allo*-ocimene (10.90%), and *α*-pinene (7.32%). Sesquiterpene hydrocarbons represented only 6.04% of the total composition, with *γ*-muurolene (3.61%) as the main metabolite of this class. Oxygenated terpenoids were totally absent, whereas the large amount of 2,4,6-trimethylbenzaldehyde (23.47%), belonging to the class “other compounds”, is noteworthy. This metabolite, quite common in *Ferulago* [38] (a genus closely related to *Cachrys*), has been never detected in any *Cachrys* species, whereas its isomer, 2,3,4-trimethylbenzaldehyde, always arising from a chemical rearrangement of ferulol derivatives [39], has been isolated in the roots of *C. sicula* [40].

The results presented here are in good agreement with those reported for another previously investigated Cretan accession of (Cc) [23]. In fact, also in this case, the main metabolite was *β*-myrcene (54.20%) and oxygenated terpenoids were absent, although it was richer in sesquiterpene hydrocarbons (36.00%). The EOs from plants collected in the Greek mainland were shown to be extremely rich in monoterpene hydrocarbons (more than 80%) [24], but in this case the content of *β*-myrcene was quite low (3.00%), with the main constituents being (*Z*)-*β*-ocimene (44.20%) and *δ*-3-carene (8.70%). A totally different profile has been reported for the EO of the Serbian accession of (Cc) [25]. In fact, it was totally devoid of monoterpene hydrocarbons and rich in sesquiterpene compounds (78.60%).

### 3.2. Detection of ROS and Antioxidant Activity Enzyme

**Figure 1:**
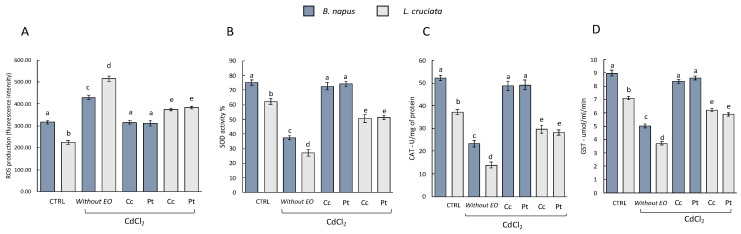
(**A**) ROS production and antioxidant/detoxifying enzyme activities (**B**) SOD, (**C**) CAT, and (**D**) GST in *B. napus* and *L. cruciate* in the control samples [i.e., without pretreatment with *Cachrys cristata* (Cc) and *Prangos trifida* (Pt) essential oils (EOs), and without treatment with CdCl_2_], in samples without pretreatment with EOs and exposed to treatment with CdCl_2_, and in samples pretreated with EOs and exposed to CdCl_2_ treatment. Bars not accompanied by the same letter (a–e) were significantly different at *p* < 0.05. Data are mean of three independent experiments ± SE (*n* = 5).

The antioxidant activity was evaluated by measuring the levels of ROS and the activity of the antioxidant enzymes SOD, CAT, and GST in the control samples (i.e., without pretreatment with EOs of (Pt) and (Cc), and without treatment with CdCl_2_), in samples without pretreatment with EOs and exposed to treatment with CdCl_2_, and in samples pretreated with EOs and exposed to CdCl_2_ treatment. As can be seen from Figure 1, samples without EOs pretreatment and exposed to CdCl_2_ treatment suffered an increase in ROS levels. In samples pretreated with EOs instead, the levels of ROS decreased. No statistically significant differences in terms of effects between the EOs of (Pt) and (Cc) used can be deduced. Both exerted a protective action with consequent reduction of ROS levels. Interestingly, however, in the EOs-pretreated *B. napus* samples, the ROS levels reached the levels measured in the control samples. This is not observed in *L. cruciata* samples, in which ROS levels decreased compared to samples without EOs pretreatment but did not reach the levels measured in control samples.

This increase in ROS in samples without pretreatment with EOs and exposed to the CdCl_2_ treatment is reflected in a decrease in the measured antioxidant enzymes [Figure 1, histograms (B), (C) and (D)]. The activity of SOD, CAT, and GST instead increased in the samples pretreated with EOs. Also in this case the protective effect of both EOs did not show statistically significant differences. However, differences in terms of response were observed with respect to the two species examined. SOD, CAT, and GST activity in *B. napus* samples pretreated with EOs was similar to the enzyme activity measured in control samples. This is not observed in *L. cruciata* samples in which SOD, CAT, and GST activity decreased compared to samples without EOs pretreatment but did not reach control levels.

In this work, for the first time the protective antioxidant effect of (Pt) and (Cc) EOs on Cd stress is investigated. This activity was tested not only on *B. napus*, often used as a “model plant”, but also on a bryophyte for environmental biomonitoring studies of heavy metal pollution. Bryophytes, considered the first divergent lineage of land plants [41], are important in studies of the response to different types of stress because they represent the earliest attempt to limit damages related to the transition from water to land, a new and inhospitable environment in which heavy metals were present due to intense geological activity.

Finally, bryophytes possess a very high surface area-to-volume ratio, have a high cation exchange capacity, do not develop strong hydrophobic barriers, and consequently are prone to “uncontrolled” metal uptake [42,43,44,45]. Therefore, also due to their wide geographical distribution, bryophytes have been used as an important biological monitoring system for metal pollution [46]. For this reason, one of the two plants chosen in this study was a bryophyte.

On the other hand, *B. napus* is a plant widely used as a superior “model” plant. It is possible to explain the reason for the different resistance to Cd damage and, consequently, the different abilities to restore the initial conditions in the two different organisms; it could be hypothesized that *B. napus* has evolved more effective defense and recovery mechanisms than those present in an ancient land plant.

The results obtained from this analysis are in line with our previous work in which the antioxidant and protective action of the EO of *Thymus leucotrichus* was demonstrated through a reduction of the ROS content (with a decrease of 1.52% and by 5.00%) and by the increase in the activity of antioxidant enzymes such as SOD (with an increase of 1.44% and 2.29%), CAT (1.46% and 2.91%), and GST (1.57% and 1.90%) in *L. riparium* samples exposed to CdCl_2_ stress [10]. These results, together with those obtained in this study, clearly indicate a possible protective capacity of EO in modulating the redox state through the antioxidant pathway by reducing Cd-induced oxidative stress.

### 3.3. Comet Assay

**Figure 2:**
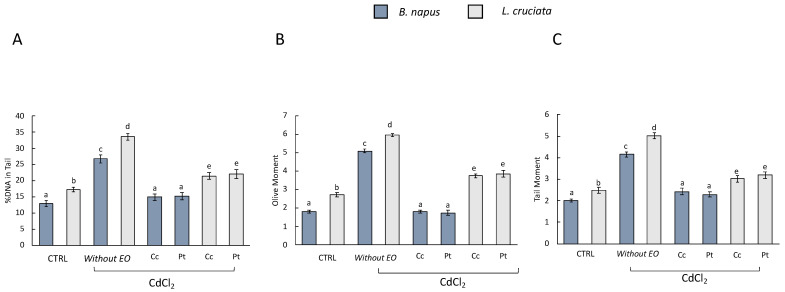
Comet assay results (**A**) DNA damage, (**B**) Olive moment, and (**C**) Tail moment in *B. napus* and *L. cruciata* in the control samples [i.e., without pretreatment with EOs of (Pt) and (Cc) and without treatment with CdCl_2_], in samples without pretreatment with EOs and exposed to treatment with CdCl_2_, and in samples pretreated with EOs and exposed to CdCl_2_ treatment. Bars not accompanied by the same letter (a–e) were significantly different at *p* < 0.05. Data are mean of three independent experiments ± SE (*n* = 5).

Cadmium-induced DNA damage was evaluated by Comet Assay. The damage was evaluated considering the following as parameters: DNA damage, Tail moment, and Olive moment in the control samples [i.e., without pretreatment with EOs of (Pt) and (Cc) and without treatment with CdCl_2_], in samples without pretreatment with EOs and exposed to treatment with CdCl_2_, and in samples pretreated with EOs and exposed to CdCl_2_ treatment (Figure 2).

In samples without EOs pretreatment and exposed to CdCl_2_ treatment, DNA damage was observed for all three parameters. In samples pretreated with EOs, on the other hand, DNA damage decreased. The EOs of (Pt) and (Cc) exerted a protective action with consequent reduction of DNA damage. Interestingly, however, in EOs-pretreated *B. napus* samples, DNA damage was comparable to that observed in control samples. This was not observed in *L. cruciata* samples, where DNA damage was decreased compared to samples without EOs pretreatment but not compared to control samples. Many scientific works have reported the healing properties of EOs based on their various biological activities. However, there is little knowledge about the protective effect of EOs against stress from pollutants and heavy metals. It has been reported that EOs had genoprotective effects against oxidative and methylating damage, which were assessed using the comet assay in HT-29 colorectal adenocarcinoma cells [47]. In addition, the EO from *Hyssopus officinalis* L. significantly reduced DNA damage in human whole blood cells, which was induced by pretreatment with hydrogen peroxide [48].

On the other hand, it was also reported that EOs can cause induction of apoptosis and DNA damage. For example, *Piper gaudichaudianum* EO treatment caused dose-dependent cytotoxic effects in V79 cells by using clonal survival, 3-(4,5-dimethylthiazole-2-yl)-2,5-biphenyl tetrazolium bromide reduction assay (MTT), and trypan blue exclusion assay, with a significant decrease in survival observed at concentrations of 0.5 μg/mL, and DNA strand breaks in V79 cells at concentrations up to 2 μg/mL as detected by the alkaline comet assay; however, the EO treatment did not induce double-strand breaks, as verified by the neutral comet assay [49].

In one previous work [10] it was demonstrated how the EO of *T. leucotrichus* counteracted the oxidative stress induced by CdCl_2_ and consequently limited the DNA damage on aquatic moss *L. riparium*. The protective capacity of the EOs of (Pt) and (Cc) in *B. napus* and *L. cruciata* is probably due to their main constituents, which belong to the same chemical classes as monoterpene hydrocarbons, sesquiterpene hydrocarbons, and oxygenated monoterpenes; the presence of these compounds conferred antioxidant, antibacterial, and antifungal activity to the EOs in different cellular and animal models.

Although the two EOs presented a difference in the composition of the individual metabolites, the biological effects shown are qualitatively identical; therefore, it is difficult to clarify and identify the real mechanism of action and the synergistic and antagonistic aspects of the individual components. But this study—about the protective effects of EOs on the stress induced by one of the most toxic heavy metals that can be introduced into the environment by human activities—is especially important from an application point of view as the first “step” in the construction of any “nutraceuticals” or various pharmaceutical preparations that can perform a protective action against oxidative stress induced by heavy metal environmental pollution.

## 4. Conclusions

Species of the genus *Prangos* and *Cachrys* can be used and idealized as a source of EOs with varied compositions that can be used for therapeutic purposes. In this scientific work, the chemical and biological properties of two different EOs isolated from Italian plants of *Prangos trifida* (Pt) and Greek plants of *Cachrys cristata* (Cc) were investigated. From the analyses carried out by GC-MS, it can be observed that both EOs are mainly dominated by the presence of hydrocarbon monoterpenes. While (Cc) EO was rich in *β*-myrcene and 2,4,6-trimethylbenzaldehyde, the EO of (Pt) was characterized by moderate amounts of sylvestrene, *α*-phellandrene, and (*Z*)-*β*-ocimene. Furthermore, in this work the protective capacity of both EOs in *L. cruciata* and *B. napus* exposed to CdCl_2_ toxicity was studied, through the study of DNA damage and the activity of antioxidant enzymes. Despite the differences in the chemical composition of the EOs, no difference in the performance of the protective action was found. Indeed, both EOs showed the ability to reduce Cd-induced oxidative stress through the restoration of antioxidant enzymatic activities and the decrease in DNA damage. What has emerged, however, is the difference between the two species examined. In fact, *B. napus* proved to be more resistant than *L. cruciate*. The different abilities to restore the initial conditions in the two different organisms could be explained by the fact that *B. napus* has evolved more effective defense and recovery mechanisms over time than those present in an ancient terrestrial plant.

This research could represent a pilot study to be used as a “model” for subsequent studies. In fact, considering the demonstrated antioxidant activity and the absence of toxic effects, including toward the bryophyte used as a model organism and toward the food plant *B. napus*, it is possible to hypothesize a possible application of essential oils for the protection of large crops grown under heavy metal stress conditions, extending the study to both other plants and other heavy metals.

## Figures and Tables

**Table 1:** Chemical composition (%) of *Cachrys cristata* (Cc) and *Prangos trifida* (Pt) essential oils (EOs).

No.	Compounds ^a^	LRI ^b^	LRI ^c^	Area (%)	
				(Cc)	(Pt)
1	*α*-Pinene	1008	1014	7.32	8.85
2	Camphene	1073	1075	-	0.24
3	Sabinene	1107	1115	0.23	0.67
4	2-Thujene	1110	1113	-	0.44
5	*β*-Pinene	1121	1125	-	1.84
6	*δ*-3-Carene	1147	1158	0.30	-
7	*β*-Myrcene	1165	1176	45.34	2.72
8	Sylvestrene	1169	1177	-	11.32
9	*α*-Phellandrene	1179	1175	-	12.14
10	*α*-Terpinene	1182	1183	-	0.26
11	(*E*)-*β*-Ocimene	1225	1233	-	3.29
12	(*Z*)-*β*-Ocimene	1242	1243	3.44	18.12
13	*allo*-Ocimene	1396	1402	10.90	1.81
14	*p*-Mentha-1,3,8-triene	1400	1408	-	9.56
15	*α*-Copaene	1504	1499	-	0.38
16	1,5,5-Trimethyl-6-methylene-cyclohexene	1553	-	0.10	
17	*α*-Bergamotene	1569	1567	-	0.76
18	*β*-Caryophyllene	1602	1612	1.35	2.74
19	*cis*-Verbenol	1649	1663	-	0.27
20	*γ*-Elemene	1629	1636	2.11	-
21	Alloaromadendrene	1659	1661	0.12	-
22	Germacrene D	1665	1687	-	5.53
23	*γ*-Muurolene	1694	1704	3.61	-
24	*δ*-Cadinene	1713	1733	0.12	-
25	Zingiberene	1715	1726	-	5.32
26	*β*-Sesquiphellandrene	1768	1776	-	2.01
27	*cis*-Sabinol	1779	1782	-	0.68
28	2,6-Dimethyl-3,5,7-octatriene-2-ol	1824	1830	-	0.73
29	2,4,6-Trimethylbenzaldehyde	1912	1929	23.47	-
30	6-Hydroxymethyl-2,3-dimethylphenyl(methanol)	1914	1918	0.48	-
31	Caryophylene oxide	2003	2008	-	0.53
32	Spathulenol	2129	2136	-	0.78
33	Phytol	2613	2622	-	0.31
	Monoterpene Hydrocarbons			67.63	71.26
	Oxygenated Monoterpenes			-	1.68
	Sesquiterpene Hydrocarbons			6.04	16.74
	Oxygenated Sesquiterpenes			-	1.31
	Others			23.95	0.31
	Total			97.62	91.30

^a^ Components listed in order of elution on a DB-Wax column; ^b^ (LRI) Linear retention index on a DB-Wax polar column; ^c^ (LRI) Linear retention indices based on literature (https://webbook.nist.gov/ accessed on 16 December 2022).

## Data Availability

Not applicable.
